# Primary care practice-based interventions and their effect on participation in population-based cancer screening programs: a systematic narrative review

**DOI:** 10.1017/S1463423623000713

**Published:** 2024-02-12

**Authors:** Ebony J. Verbunt, Grace Newman, Nicola S. Creagh, Kristi M. Milley, Jon D. Emery, Margaret A. Kelaher, Nicole M. Rankin, Claire E. Nightingale

**Affiliations:** 1 Centre for Health Policy, Melbourne School of Population and Global Health, The University of Melbourne, Melbourne, VIC, Australia; 2 Centre for Cancer Research and Department of General Practice, Victorian Comprehensive Cancer Centre, University of Melbourne, Melbourne, VIC, Australia

**Keywords:** bowel cancer screening, breast cancer screening, cervical screening, practice-based interventions, primary care, primary healthcare workers

## Abstract

**Aim:**

To provide a systematic synthesis of primary care practice-based interventions and their effect on participation in population-based cancer screening programs.

**Background:**

Globally, population-based cancer screening programs (bowel, breast, and cervical) have sub-optimal participation rates. Primary healthcare workers (PHCWs) have an important role in facilitating a patient’s decision to screen; however, barriers exist to their engagement. It remains unclear how to best optimize the role of PHCWs to increase screening participation.

**Methods:**

A comprehensive search was conducted from January 2010 until November 2023 in the following databases: Medline (OVID), EMBASE, and CINAHL. Data extraction, quality assessment, and synthesis were conducted. Studies were separated by whether they assessed the effect of a single-component or multi-component intervention and study type.

**Findings:**

Forty-nine studies were identified, of which 36 originated from the USA. Fifteen studies were investigations of single-component interventions, and 34 studies were of multi-component interventions. Interventions with a positive effect on screening participation were predominantly multi-component, and most included combinations of audit and feedback, provider reminders, practice-facilitated assessment and improvement, and patient education across all screening programs. Regarding bowel screening, provision of screening kits at point-of-care was an effective strategy to increase participation. Taking a ‘whole-of-practice approach’ and identifying a ‘practice champion’ were found to be contextual factors of effective interventions.

The findings suggest that complex interventions comprised of practitioner-focused and patient-focused components are required to increase cancer screening participation in primary care settings. This study provides novel understanding as to what components and contextual factors should be included in primary care practice-based interventions.

## Introduction

The success of population-based cancer screening programs in reducing cancer mortality is often limited by sub-optimal participation in the community. Fifty percent of the eligible population participate in Germany’s Mammography Screening Program (Hand, 2020), while less than half (44%) participate in Australia’s National Bowel Cancer Screening Program (Australian Instititute of Health and Welfare, 2020). Scotland and Canada have participation rates between 60% and 81% for their breast and cervical screening programs. However, lower participation rates are observed in these countries' bowel screening programs (McCowan *et al.*, 2019; Ontario Health, 2021). Many patient-level barriers have been described, including low awareness of program existence (Ferdous *et al.*, 2018; Suwankhong and Liamputtong, 2018), worry about the procedure or outcome (Ferdous *et al.*, 2018; Suwankhong and Liamputtong, 2018; Muthukrishnan *et al.*, 2019), and time and transport required to attend screening (Suwankhong and Liamputtong, 2018; Muthukrishnan *et al.*, 2019). People can be encouraged to engage with screening programs through invitations (Radde *et al.*, 2016), small and mass media campaigns (Durkin *et al.*, 2019; Schliemann *et al.*, 2019), and through other prompts, such as celebrity cancer diagnosis, which have been shown to result in increased screening appointments and call to helplines (Boudioni *et al.*, 1998; Chapman *et al.*, 2005). Importantly, endorsement of screening by a primary healthcare worker (PHCW) is an important facilitator in a patient’s decision to screen (Duffy *et al.*, 2017). However, PHCWs often experience challenges in engaging in screening programs, with barriers including the financial structure of primary care, the structure of screening programs, time, and screening knowledge (Wender, 1993; Yarnall *et al.*, 2003; Verbunt *et al.*, 2022).

The role of PHCWs, including family physicians/general practitioners (GPs)^1^ and practice nurses, in cancer screening programs differs between countries and programs. Accredited PHCWs are responsible for facilitating screening tests in most cervical screening programs (Fontham *et al.*, 2020; Australian Government Department of Health, 2021b). As mammography screening mostly occurs outside of primary care, PHCWs play a less direct but important role in promoting participation and facilitating follow-up care (Klarenbach *et al.*, 2018; The Royal Australian College of General Practitioners, 2018). For bowel cancer screening, the role of PHCWs differs according to the program structure. In the US and Canada, family physicians are responsible for recommending, performing, or referring patients to different screening modalities, including colonoscopy and fecal occult blood test (FOBT) (Canadian Task Force on Preventive Health, 2001; Force *et al.*, 2016). However, in countries such as the United Kingdom and Australia, eligible participants receive a fecal immunochemical test or FOBT screening kit via mail and have the option to nominate their GP to receive results and provide follow-up care (National Health Service, 2021; Australian Government Department of Health, 2021a).

Reviews, although not systematic, exist on the effect of interventions addressing practitioner-level barriers to engaging in cancer screening. A 2012 review concluded that the engagement of PHCWs and screening participation could be improved by using audit and feedback systems and office-system prompts, such as reminders for the clinician to discuss or order cancer screening tests (Emery *et al.*, 2012). A more recent review found interactive and multi-faceted continuous medical education, training with audit and feedback, enablement through IT-based systems, and collaborative team-based interventions can modify PHCW’s practice and improve patient outcomes (Chauhan *et al.*, 2017). However, this review was not specific to cancer screening, nor did it discuss any magnitude of effect on cancer screening participation. Further, contextual factors, defined as the ‘features of the circumstances in which an intervention is implemented that may interact with the intervention to produce variation in outcomes’, were not outlined (Craig *et al.*, 2018). Contextual factors, such as workload and leadership, are an important consideration for understanding how, and under what circumstances interventions create change (Moore *et al.*, 2015; Skivington *et al.*, 2021).

Thus, it is unclear how to best optimize the role of PHCWs to increase cancer screening participation. This review aims to provide a systematic synthesis of primary care practice-based interventions and their effect on participation in population-based cancer screening programs. Contextual factors of effective interventions will be summarized. Findings from this review can be used to guide the development of interventions using PHCWs to facilitate greater participation in bowel, breast, and cervical screening programs.

## Methods

The review is structured in accordance with the Preferred Reporting Items for Systematic Reviews and Meta-Analyses statement (Page *et al.*, 2021). The review protocol was registered with the International Prospective Register for Ongoing Systematic Reviews; CRD42020201118. All components of the protocol were adhered to; however, for clarity purposes, the outcome of interest was limited to screening uptake. Search terms were developed with the assistance of a research librarian (Appendix 1). Medline (OVID), EMBASE, and CINAHL were searched for articles published from 1st January 2010 to 23rd November 2023. Reference lists of included articles were searched for additional studies.

### Study selection

#### Inclusion and exclusion criteria

Articles were included if they met the following criteria: (1) Randomized controlled trials (RCTs), non-randomized trials; (2) Intervention conducted in a high-income country (The World Bank, 2022); (3) Focused on the effect of a primary care practice-based intervention(s) to optimize the role of PHCWs in population-based cancer screening program (bowel, breast, and cervical); (4) Measures screening participation as an outcome; and (5) Published in English. Articles were excluded if they met any of the following criteria: (1) Reviews, protocols, and conference abstracts; (2) Impact of the intervention measured outside of a primary care setting; (3) Not focused on primary care practice-based intervention(s) to optimize the role of PHCWs in population-based cancer screening programs (bowel, breast, and cervical); (4) Intervention not targeted at a PHCW; and (5) Screening participation not included as an outcome.

Search results were imported into Covidence (Babineau, 2014) and duplicates were excluded. Two authors (EV, NC) independently screened titles and abstracts for relevance and three authors (EV, NC, CN) independently assessed the eligibility of full-text articles. The authors discussed disagreements to reach a consensus.

#### Data extraction and synthesis

A data extraction tool was developed for the review and piloted independently by two authors (EV, CN) who met several times to reach a consensus on the final information to be extracted. Three authors (EV, GN, CN) independently extracted: authors; year of publication; country; screening program; study type; population; sample size; intervention components; comparison; follow-up time; effect on screening participation; and contextual factors of intervention. Contextual factors that were explicitly outlined in studies surrounding primary care practice-based interventions were extracted.

Intervention components were categorized as practice-focused or patient-focused, with each category divided into sub-categories (Table 1). Because of the heterogeneity of interventions and study designs, we did not undertake meta-analysis, instead using narrative synthesis to summarize data (Popay *et al.*, 2006). Studies were categorized by whether they assessed the effect of a single-component or multi-component intervention and study type.

**Table 1.** Practice-focused and patient-focused intervention components

Practice-focused	
Component of intervention	Definition
Audit and feedback	A systematic collection of data on cancer screening professional performance based on criteria or standards subsequently fed back to practitioners in a structured manner
Reminders	Paper or electronic reminders to practitioners to discuss cancer screening with patients or to conduct cancer screening
Financial incentives	A practitioner or practice payment received for engaging patients in a screening test or for participating in a cancer screening intervention
Education and/or training	Improving a practitioner’s knowledge, such as understanding of cancer screening guidelines or skills, such as the ability to communicate effectively with patients
Behavior change techniques	Implementing techniques, such as anticipated regret, which are designed to change a practitioner’s behavior
Practice-facilitated assessment and improvement	Based on a review of current practice activities, multiple practice activities are undertaken to facilitate the delivery of cancer screening

Patient-focused	
Reminders	Paper or electronic reminders to patients that they are due or overdue for cancer screening
Education	Improving patient’s knowledge, such as on cancer screening guidelines and the benefits of cancer screening
Patient navigation	Support provided to a patient to navigate the cancer screening process
Promotional material	Posters or other materials promoting cancer screening to patients, usually placed in the waiting room of a primary care setting
Financial incentives	The patient receives payment for participating in cancer screening
Provision of screening kit	The patient receives a bowel cancer screening kit at point-of-care
Letter of recommendation	Patients receive a letter from their practitioner recommending cancer screening

#### Quality assessment

To assess the quality of randomized and non-randomized studies, the Downs and Black Checklist was used (Downs and Black, 1998). The checklist has 27 questions within five categories: reporting [10 items], external validity [3 items], internal validity/bias [7 items], internal validity/confounding [6 items], and power [1 item]. Item 27 (power) was modified. Studies were rated on whether or not they performed an a priori power calculation, with the maximum score for this item being 1 rather than 5. The highest possible score for the checklist was therefore 28 instead of 32. Two authors (EV, NC) independently assessed each article, meeting to discuss and resolve discrepancies. Articles were rated as being of excellent (26–28); good (20–25); fair (15–19); or poor (≤14) quality (Hooper *et al.*, 2008).

## Results

### Study selection

**Figure 1.**
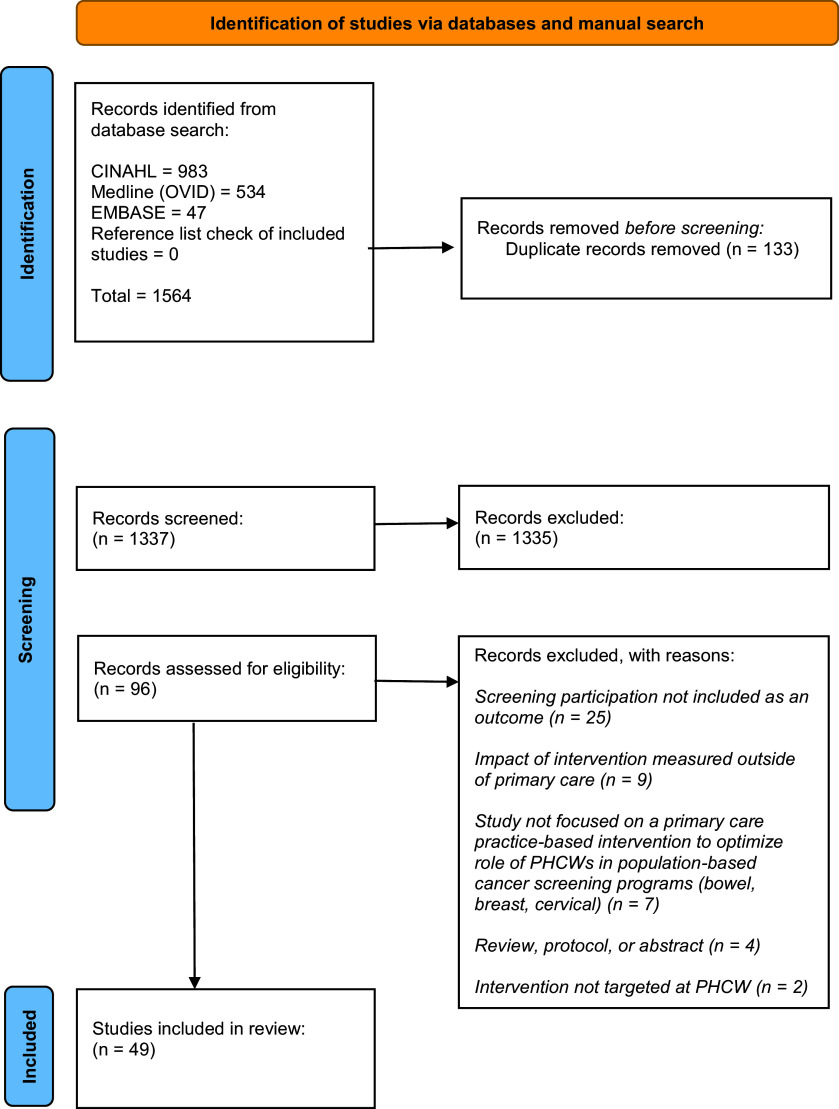
Prisma diagram of study selection

We identified 1564 studies, with 133 duplicate studies removed before screening. Following title and abstract screening, we excluded 1335 studies, leaving 96 studies for full-text screening. Following full-text screening, we excluded 47 studies (Fig. 1), with 49 studies therefore included in our systematic synthesis. No additional studies were found when searching the reference lists of included studies.

### Critical appraisal

Overall, RCTs had a mean score of 22 (good) and non-randomized trials 17 (fair). RCTs had a higher mean score than non-randomized trials in internal validity/confounding (4 versus 2), with similar mean scores in the remaining methodological categories – reporting (9 versus 8), external validity (3 versus 3), and internal validity/bias (4 versus 4) (Appendix 2).

### Study characteristics

#### Characteristics of studies

**Figure 2.**
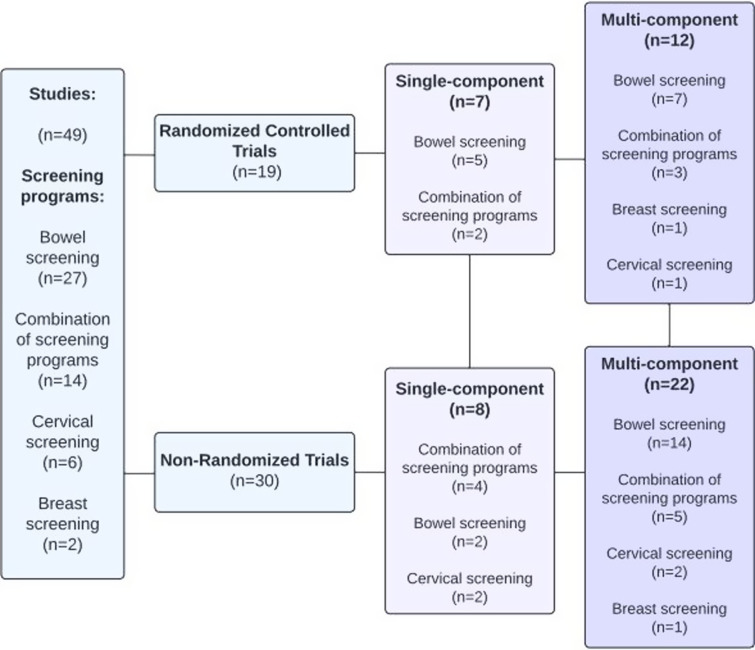
Characteristics of studies.

Studies originated from the US (*n* = 36), Canada (*n* = 5), Australia (*n* = 3), France (*n* = 2), Norway (*n* = 1), Netherlands (*n* = 1), and Spain (*n* = 1). Study designs included RCTs (*n* = 19) and non-randomized trials (*n* = 28). Interventions focused on increasing participation in bowel screening (*n* = 27), six targeted cervical screening, two breast screening, and 14 studies focused on a combination of all three screening programs. Fifteen studies were investigations of single-component interventions, including seven RCTs, and eight non-randomized trials. Thirty-four studies included multi-component interventions, of which 12 were RCTs, and 22 were non-randomized trials (Fig. 2).

### Single-component – RCTs

Seven studies from the US (*n*  = 3), France (*n*  = 2), Spain (*n*  = 1), and Canada (*n*  = 1) were single-component RCTs (Dignan *et al.*, 2014; Aubin-Auger *et al.*, 2016; Guiriguet and Castells, 2016; Rat *et al.*, 2017; Wang *et al.*, 2018; Hwang *et al.*, 2019; Vaisson *et al.*, 2019) (Table 2).

**Table 2.** Overview of single-component primary care practice-based interventions and effect on cancer screening participation

First author, year, country	Practice-focused intervention category	Cancer	Comparator	Follow-up period (month/s)	Effect measure	Findings* and estimates	Contextual factor(s)	Quality score – rating
**Single-component RCTs**								
Aubin-Auger, 2016, France	Training	Bowel	Usual care	7	Intervention group versus Control group	**No effect** 36.7% versus 24.5%, *P* = 0.03	n/a	22 – good
Dignan, 2014, US	Training	Bowel	Delayed intervention	6	Intervention group versus Control group	**No effect** 79.7% versus 71.2%, *P* = 0.06	n/a	19 – fair
Wang, 2018, US	Training	Bowel	Usual care	6	Intervention group versus Control group	**No effect** 24.4% versus 17.7%, *P* = 0.24	n/a	24 – good
Guiriguet, 2016, Spain	Reminders	Bowel	Usual care	12	Intervention group versus Control group	**No effect** 44.1% versus 42.2%, *P* = 0.1466	n/a	23 – good
Rat, 2017, France	Patient-specific reminders	Bowel	Usual care	12	Difference between trial arms	**Positive effect** 4.2% (95% CI, 2.3 to 6.2), *P* < 0.001)	n/a	21 – good
	Patient-specific reminders		Generic reminders			**Positive effect** 3.1% (95% CI, 1.3 to 5.0), *P* < 0.001		
	Generic reminders		Usual care			**No effect** 1.1% (95% CI, −0.6 to 2.8), *P* < 0.21)		
Hwang, 2019, US	Audit and feedback – practice target	Bowel	Baseline	12	Pre-Post	**Positive effect** 64.6% to 72.5%, *P* < 0.01	n/a	21 – good
	Audit and feedback -practice target	Cervical				**Positive effect** 65.30% to 77%, *P* < 0.01		
Vaisson, 2019, Canada	Behavior change technique – anticipated regret influencing use of an audit and feedback tool	Bowel	Usual care	13	Rate ratio	**No effect** 1 (95% CI, 0.998 to 1.002), *P* = 0.99	n/a	25 – good
	Behavior change technique – material incentive influencing use of an audit and feedback tool	Bowel				**No effect** 1 (95% CI, 0.998 to 1.002), *P* = 0.93		
	Behavior change technique – problem-solving influencing use of an audit and feedback tool	Bowel				**No effect** 1 (95% CI, 0.998 to 1.002), *P* = 0.95		
	Behavior change technique – anticipated regret influencing use of an audit and feedback tool	Breast				**No effect** 1 (95% CI, 0.997 to 1.002), *P* = 0.81		
	Behavior change technique – material incentive influencing use of an audit and feedback tool	Breast				**No effect** 1 (95% CI, 0.997 to 1.003), *P* = 0.94		
	Behavior change technique – problem-solving influencing use of an audit and feedback tool	Breast				**No effect** 0.998 (95% CI, 0.995 to 1.001), *P* = 0.13		
	Behavior change technique – anticipated regret influencing use of an audit and feedback tool	Cervical				**No effect** 1.002 (95% CI, 0.998 to 1.003), *P* = 0.83		
	Behavior change technique – material incentive influencing use of an audit and feedback tool	Cervical				**No effect** 1 (95% CI, 0.998 to 1.002), *P* = 0.81		
	Behavior change technique – problem-solving influencing use of an audit and feedback tool	Cervical				**Positive effect** 1.003 (95% CI, 1.001 to 1.006), *P* = 0.003		
**Single-component non-randomized trials**								
Greene, 2013, Australia	Financial incentive	Cervical	Baseline	n/a	Mixed-method evaluation	**No effect** 5% increase – not sustained	n/a	16 – fair
Kiran, 2014, Canada	Financial incentives	Bowel	Baseline	12	Step change after introduction of incentives	**No effect** 0.95 (95% CI, −2.2 to 4.1)	n/a	19 – fair
		Breast				**No effect** 1.6 (95% CI, −0.25 to 3.4)		
		Cervical				**No effect** 1.5 (95% CI, −0.80 to 3.8)		
Kirschner, 2013, Netherlands	Financial incentive	Cervical	Baseline	12	Pre-Post	**No effect** 71.90% to 72.50%	n/a	18 – fair
Gavagan, 2010, US	Financial incentive	Breast	Non-incentivized clinics	18	Linear mixed model	**No effect** Slope: 0.003 versus 0.0015, *P* = 0.076	n/a	17 – fair
		Cervical				**No effect** Slope: 0.005 versus −0.004, *P* = 0.053		
Jung, 2017, US	Financial incentive – adopting/using EHR	Bowel	Non-incentivised family physicians	12	Odds ratio	**Positive effect** 1.2 (95% CI, 1.1 to 1.4)	n/a	18 – fair
Curry, 2011, US	Education	Bowel	Baseline	6	Pre-Post	**No effect** 56% to 60%, *P* = 0.29	n/a	16 – fair
Hsiang, 2019, US	Reminders	Bowel	Control practices	12	Change in patient completion of screening	**No effect** 1.0 percentage points (95% CI, −3.2 to 4.6 percentage points)	n/a	19 – fair
		Breast				**No effect** 0.1 percentage points (95% CI, −4.0 to 4.3 percentage points)		
Jonah, 2017, Canada	Audit and feedback	Bowel – due for screening	Control family physicians	5	Odds ratio	**Positive effect** 1.08 (95% CI, 1.05 to 1.12)	n/a	18 – fair
		Bowel – overdue for screening				**Positive effect** 1.15 (95% CI, 1.12 to 1.19)		
		Breast- due for screening				**No effect** 0.98 (95% CI, 0.96 to 1.01)		
		Breast – overdue for screening				**Positive effect** 1.06 (95% CI, 1.04 to 1.09)		
		Cervical – due and overdue for screening				**Positive effect** 1.09 (95% CI, 1.07 to 1.12)		

* No effect refers to no statistically significant effect of the intervention.

#### Training and/or education

Three cluster RCTs assessed whether training and/or education for PHCWs increased bowel cancer screening participation. Aubin-Auger *et al.* (2016) assessed the effect of training focused on improving GPs’ communication skills, Dignan *et al.* (2014) investigated whether more comprehensive PHCW-focused training and education on the following topics – screening efficacy, clinical performance measures, patient counseling, and creating a screening-friendly environment increased participation, and Wang *et al.* (2018) tested whether training family physicians on patient-centered communication had an impact. None of these trials increased screening participation compared to usual care.

#### Provider reminders

Two cluster RCTs assessed whether provider reminders could increase bowel screening participation. Guiriguet and Castells (2016) evaluated the effect of an alert in electronic medical records for first-time screeners, finding no effect on screening rate when compared to the control group. Conversely, Rat *et al.* (2017) found providing GPs with a list of patients who were non-adherent to screening (patient-specific reminders) compared to usual care had a positive effect on screening rates. A positive effect on screening rates was also found when comparing patient-specific reminders to generic reminders; however, no effect was found when comparing generic reminders to usual care.

#### Audit and feedback

Two studies assessed the effect of audit and feedback on screening. Hwang *et al.* (2019) found an audit and feedback tool to have a positive effect on bowel and cervical screening rates compared to baseline. Vaisson *et al.* (2019) examined the effect of emails to providers that used behavior change techniques (either problem-solving, anticipated regret, or material incentive) to promote access to an existing audit and feedback tool. Among practitioners receiving problem-solving emails, there was a positive effect on cervical screening, but there was no effect on bowel or breast screening participation. Emails operationalizing anticipated regret and material incentives had no effect.

### Single-component – non-randomized trials

Eight studies from the US (*n*  = 5), Australia (*n*  = 1), Netherlands (*n*  = 1), and Canada (*n*  = 1) were single-component non-randomized trials (Gavagan *et al.*, 2010; Curry *et al.*, 2011; Greene, 2013; Kirschner *et al.*, 2013; Kiran *et al.*, 2014; Jonah *et al.*, 2017; Jung *et al.*, 2017; Hsiang *et al.*, 2019) (Table 2).

#### Financial incentives

Five studies assessed whether financial incentives to providers would increase screening participation of which three focused on pay-for-performance (P4P) programs. Greene (2013) examined the effect of paying GPs to conduct cervical screening for overdue women, Kiran *et al.* (2014) evaluated a P4P program with high uptake among family physicians, which offered substantial financial incentives to increase bowel, breast, and cervical screening, and Kirschner *et al.* (2013) assessed whether general practice receiving a bonus based on a score of patient care increased screening. None of these P4P programs had an impact on screening rates compared to baseline. Gavagan *et al.* (2010) found that doubling financial incentives related to achieving group targets on breast or cervical screening rates had no effect. In Jung *et al.* (2017), family physicians participated in an initiative where payments were received for adopting and using electronic health records (EHRs). Unlike the previous studies, a positive effect on bowel screening rates was found.

#### Education and training, provider reminders or audit and feedback

Three studies assessed the effect of education and training, provider reminders, or audit and feedback. Curry *et al.* (2011) examined an education and training intervention, finding no effect on bowel screening rates. Hsiang *et al.* (2019) assessed the effect of provider reminders, finding that although there was a large increase in clinician ordering of tests, there was no effect on screening rates for bowel and breast. Jonah *et al.* (2017) assessed an audit and feedback tool, finding a positive effect on screening rates among people due and overdue for bowel and cervical screening, and among patients overdue for breast screening. Among women due for breast screening, there was no change in screening rates.

### Multi-component – RCTs

Twelve studies from the US (*n*  = 10), Australia (*n*  = 1), and Norway (*n*  = 1) were multi-component RCTs (Aragones *et al.*, 2010; Ornstein *et al.*, 2010; Atlas *et al.*, 2011; Shaw *et al.*, 2013; Atlas *et al.*, 2014; Price-Haywood *et al.*, 2014; Basch *et al.*, 2015; Sun *et al.*, 2018; Dodd *et al.*, 2019; Cameron *et al.*, 2020; Moen *et al.*, 2020; Walsh *et al.*, 2020) (Table 3).

**Table 3.** Overview of multi-component primary care practice-based interventions and effect on cancer screening participation

First author, year, country	Practice-focused intervention category	Patient-focused intervention category	Cancer	Comparator	Follow-up period (month/s)	Effect measure	Findings* and estimates	Contextual factor(s)	Quality score – rating
**Multi-component RCTs**									
Price-Haywood, 2014, US	Training	n/a	Bowel	Audit and feedback	24	Intervention group (pre-post) versus Control group (pre-post)	**No effect** 23.4% to 44% versus 35.1% to 63.2%	n/a	22 – good
	Audit and feedback		Breast				**Positive effect** 22.3% to 43.3% versus 35.1% to 63.2%, *P* < 0.05		
			Cervical				**No effect** 20.2% to 34.4% versus 33.7% to 56.7%		
Shaw, 2013, US	Practice-facilitated assessment and improvement	n/a	Bowel	Control	12	Odds ratio	**No effect** 1.17 (95 CI, 0.86 to 1.59, *P* = 0.32	Whole-of-practice approach	24 – good
	Education and training							Practice champion	
Ornstein, 2010, US	Audit and feedback	n/a	Bowel	Control	24	Difference between trial arms	**Positive effect** 4.9% (95% CI, 3.6 to 6.1), *P* < 0.0001	Whole-of-practice approach	23 – good
	Education and training								
Aragones, 2010, US	Reminder	Promotional material	Bowel	Usual care	3	Odds ratio	**Positive effect** 5.4 (95% CI, 1.6 to 18.5)	n/a	24 – good
		Education							
Moen, 2020, Norway	Education	Promotional material	Cervical	Usual care	6	Odds ratio	**Positive effect** 1.24 (95% CI, 1.11 to 1.38)	n/a	20 – good
	Reminder								
Walsh, 2020, US	Reminder	Promotional material	Bowel	Usual care	14	Intervention group versus Control group	**No effect** 17.9% versus 11.6%, *P* = 0.31	n/a	21 – good
			Breast				**No effect** 4% versus 6.4%, *P* = 0.48		
			Cervical				**No effect** 10.7% versus 13.9%, *P* = 0.89		
Atlas, 2011, US	Reminder	Reminder	Breast	Usual care	12	Intervention group versus Control group	**Positive effect** 31.4% versus 23.2%, *P* < 0.001	Whole-of-practice approach	21 – good
		Patient navigation							
Atlas, 2014, US	Reminder	Reminder	Bowel	Patients receiving reminder and transfer to practice delegate lists	12	Intervention group versus Control group	**No effect** 77.8% versus 76.2%, *P* = 0.33	Whole-of-practice approach	24 – good
		Patient navigation	Breast				**No effect** 82.7% versus 82.7% *P* = 0.96		
			Cervical				**No effect** 84.1% versus 84.7%, *P* = 0.6		
Dodd, 2019, Australia	Training	Education	Bowel	Usual care	1.5	Odds ratio	**Positive effect** 10.24 (95% CI, 2.9 to 36.6), *P* = 0.0006	n/a	20 – good
		Provision of screening kit							
Sun 2018, US	Education	Education	Bowel	Usual care	12	Intervention group versus Control group	**Positive effect** 58.5% versus 22.2%, *P* < 0.0001	n/a	17 – fair
		Provision of screening kit							
		Letter of recommendation							
Basch, 2015, US	n/a	Education	Bowel	Provider education	12	Difference between trial arms	**No effect** 18.3% versus 20.0%, *P* = 0.79	n/a	22 – good
	Education	n/a		Provider education and patient education			**No effect** 20% versus 25.6%, *P* = 0.23		
	n/a	Education		Provider education and patient education			**No effect** 18.3% versus 25.6%, *P* = 0.11		
Cameron, 2020, USA	Education and training Audit and feedback	Promotional material Education	Bowel	Usual care	6	Rate ratio	**No effect** 1.65 (95% CI, 0.62 to 4.38, *P* = 0.32)	n/a	23 – good
	Education and training Audit and feedback	n/a		Patient promotional material and patient education			**No effect** 0.80, (95% CI, 0.53 to 1.20, *P* = 0.28)		
**Multi-component non-randomized trials**									
Ruggeri, 2020, US	Practice-facilitated assessment and improvement	Education	Bowel	Baseline	12	Pre-Post	**Positive effect**	n/a	15 – fair
							Practice 1 = 32.3% to 45.4%		
		Provision of screening kit					Practice 2 = 27.7% to 36.8%		
							Practice 3 = 23.2% to 47.9%		
	Education	Reminder					Practice 4 = 27.5% to 36.8%		
Hills, 2015, US	Practice-facilitated assessment and improvement	Reminder	Cervical	Baseline	12	Pre-Post	**Positive effect** 38.1% to 69.7%, *P* < 0.001	Whole-of-practice approach	16 – fair
	Education							Practice champion	
Walker-Smith, 2020, US	Practice-facilitated assessment and improvement	n/a	Breast	Baseline	3	Pre-Post	**Positive effect** 54.8% to 65.2%	n/a	17 – fair
	Training								
Frissora, 2021, US	Practice-facilitated assessment and improvement	Education	Bowel	Baseline	5	Pre-Post	**Positive effect** 69.8% to 76%	n/a	16 – fair
	Education								
	Audit and feedback								
Desai, 2021, US	Practice-facilitated assessment and improvement	Education	Bowel	Baseline	3	Pre-Post	**Positive effect**	Practice champion	16 – fair
							Practice 1 = 41% to 48.4%, *P* < 0.0001		
							Practice 2 = 31.6% to 37.8%, *P* < 0.0001		
							Practice 3 = 30.5% to 38.2%, *P* < 0.0001		
							Practice 4 = 43.9% to 46.8%, *P* = 0.012		
Hussain 2021, US	Practice-facilitated assessment and improvement	Promotional material	Bowel	Pre-intervention group	n/a	Post-Group versus Pre-Group	**Positive effect** 74.4% (95% CI, 64.6 to 82.3) versus 31.1% (95% CI, 26.2 to 36.6, *P* < 0.001	Whole-of-practice approach	18 – fair
	Training	Reminders							
Weiner, 2017, USA	Practice-facilitated assessment and improvement	Provision of screening kit	Bowel	Baseline	12	Pre-Post	**Positive effect** 23.0% to 34.0%, *P* = 0.03	Whole-of-practice approach	17 – good
								Practice champion	
	Reminder								
	Financial incentive								
Mader, 2016, US	Practice-facilitated assessment and improvement	n/a	Bowel	Baseline	6	Pre-Post	**Positive effect** 32.74% to 38.3%, *P* < 0.001	Whole-of-practice approach	17 – fair
	Education		Breast				**Positive effect** 36.96% to 49.96%, *P* < 0.001	Practice champion	
			Cervical				**Negative effect** 35.65% to 38.95%, *P* = 0.998		
Jones 2022, US	Practice-facilitated assessment and improvement Audit and feedback	Reminders	Bowel	Pre-intervention group	12	Prevalence ratio	**Positive effect** 1.85 (95% CI, 91.63 to 2.11), *P* < 0.05	Whole-of-practice approach	19 – fair
			Breast				**No effect** 1.11 (95% CI, 0.98 to 1.27), *P* > 0.05	Practice champion	
			Cervical				**Positive effect** 1.44 (95% CI, 1.18 to 1.76), *P* < 0.05		
Nguyen, 2020, US	Practice-facilitated assessment and improvement Education Financial incentives	n/a	Bowel	Pre-intervention group	24	Difference-in-differences	**No effect** −0.2 (−1.64, 1.62), *P* = .99	Whole-of-practice approach	15 – fair
			Breast				**No effect** 1.80 (−0.87, 4.47), *P* = .18		
			Cervical				**No effect** −2.56 (−5.44, 0.31), *P* = 0.08		
Harris, 2015, Canada	Practice-facilitated assessment and improvement Education and training	Education	Bowel	Control	24	Intervention group versus Control group	**No effect** 66.1% versus 61.7%, *P* = 0.77	Whole-of-practice approach	20 – good
Green, 2017, Canada	Practice-facilitated assessment and improvement Education and training	Education	Bowel	Baseline	12–24	Difference between trial arms	**Positive effect** 5.4% (95% CI, 3.1 to 7.8)	n/a	20 – good
			Cervical				**Positive effect** 2.7% (95% CI 0.9 to 4.6)		
Marx, 2016, US	Practice-facilitated assessment and improvement Audit and feedback Education and training Financial incentives	Education Reminders	Bowel	Baseline	n/a	Change in screening prevalence	**Positive effect** 1.1% to 14.5% (3 clinics)	n/a	16 – fair
Dorrington, 2015, Australia	Practice-facilitated assessment and improvement	Promotional material	Cervical	Baseline	10	t-tests	**Positive effect** t(50) = −3.221, *P* = 0.002	n/a	18 – fair
	Education								
Hountz, 2017, US	Practice-facilitated assessment and improvement	Promotional material	Bowel	Baseline	11	Pre-post	**Positive effect** 30% to 58%, *P* < 0.001	Whole-of-practice approach	16 – fair
	Reminders								
Bakhai, 2018, US	Practice-facilitated assessment and improvement	Patient navigation	Bowel	Baseline	6	Pre-post	**Positive effect** 50% to 75%	Whole-of-practice approach	16 – fair
	Reminder	Education						Practice champion	
	Education	Financial incentive							
Baxter, 2017, Canada	4–5 components, including – Reminder	n/a	Bowel	0–1 practice-focused components	24	Hazard ratio	**Positive effect** 1.27 (95% CI, 1.16 to 1.39, *P* < 0.0001)	n/a	19 – fair
	Audit and feedback								
Potter, 2011, US	Education and training	Education	Bowel	Usual care	12	Difference between trial arms	**Positive effect** 18% versus 1.7%, *P* < 0. 001	n/a	16 – fair
		Provision of screening kit							
Funes, 2021, US	Education and training	Reminder	Bowel	Usual care	12	Odds ratio	**Positive effective** 2.4 (95% CI, 1.6 to 2.6), *P* = .001	n/a	16 – fair
		Provision of screening kit							
Wu, 2016, US	Reminder	Reminder	Bowel	Usual care	5	Intervention group versus control group	**Positive effect** 81.0% versus 78.1%, *P* < 0.05	Whole-of-practice approach	17 – fair
		Education						Practice champion	
		Patient navigation							
Willemse, 2022, US	Education	Education	Bowel	Baseline	5	Pre-post	**Positive effect** 45.8% to 65.4%	n/a	15 – fair
		Reminder							
		Provision of screening kit							
Kaczorowski, 2013, Canada	Financial incentives	Reminder	Breast	Baseline	2	Pre-post (difference in screening rates)	**Positive effect** 6.3% (*P* < 0.001)	n/a	18 – fair
	Reminder		Cervical				**Positive effect** 5.3% (*P* < 0.001)		

* No effect refers to no statistically significant effect of the intervention.

#### Practitioner-focused intervention components

Three studies assessed the effect of interventions with multiple practitioner-focused components on screening rates. Price-Haywood *et al.* (2014) looked at whether training family physicians on how to engage in cancer risk communication, in addition to audit and feedback, would increase screening. When compared to audit-only, there was a positive effect on breast screening, but no effect on bowel and cervical screening rates. Shaw *et al.* (2013) found no effect on bowel screening rates when comparing practices participating in practice-facilitated assessment and improvement and education and training to control practices. However, Ornstein *et al.* (2010) found a positive effect on bowel screening rates when assessing the impact of audit and feedback and education and training compared to control practices.

#### Provider reminders and patient promotional material

Three studies evaluated the effect of interventions involving provider reminders and patient promotional material in the waiting room compared to usual care. In Aragones *et al.* (2010), patients watched a bowel screening video and received a brochure summarizing the video’s messages. They were also given a reminder to hand it to their family physician. A positive effect on bowel screening rates was found. Similarly, Moen *et al.* (2020) found a positive effect on cervical screening rates following an educational session for GPs, provider reminders, and promotional material in the form of a poster. Walsh *et al.* (2020) assessed the effect of promotional material, in the form of a virtual ‘Doctor’ and provider reminders. However, unlike Aragones *et al.* (2010) and Moen *et al.* (2020), no effects on bowel, breast, or cervical screening rates were found.

#### Audit and feedback, patient reminders, and patient navigation

Two studies assessed the effect of audit and feedback, patient reminders, and patient navigation. Atlas *et al.* (2011) evaluated the use of an informatics system, which connected overdue patients to appropriate providers and presented providers with a list of their overdue patients. Patients received reminders and navigation. A positive effect on breast screening rates was found when compared to usual care. More recently Atlas *et al.* (2014) studied the impact of involving family physicians in a web-based IT application, with physicians in the intervention arm receiving a list of their overdue patients and providing individualized outreach – via a letter, practice delegate, or practice navigator. However, in comparison to the control, where overdue patients were automatically sent reminder letters and transferred to patient delegate lists, no effect on bowel, breast, or cervical screening rates was found.

#### Practitioner-focused and patient-focused intervention components

Two studies assessed the effect of interventions comprising different combinations of practitioner-focused and patient-focused components, with both studies including patient education and provision of a bowel cancer screening kit at point-of-care. Dodd *et al.* (2019) assessed the effect of providing GPs with a script to assist them in endorsing screening and providing patients with a screening kit and education. Similarly, in Sun *et al.* (2018), family physicians received education and patients received education, a screening kit, and a letter of recommendation from their physician. Both studies reported a positive effect on bowel screening rates.

#### Difference between practitioner-focused and/or patient-focused intervention components

Two studies assessed whether there was a difference between intervention groups in screening rates. Basch *et al.* (2015) compared screening rates between – 1. patients receiving educational material, 2. family physicians receiving education, and 3. family physicians receiving education and patients also receiving education. Cameron *et al.* (2020) compared bowel screening rates between – 1. Physicians receiving education and audit and feedback and patients watching an educational video, 2. Physicians receiving education and audit and feedback, and 3. Usual care. No effect on bowel screening rates was found for either study.

### Multi-component – non-randomized trials

Twenty-two studies from the US (*n*  = 18), Canada (*n*  = 3) and Australia (*n*  = 1) were multi-component non-randomized trials (Potter *et al.*, 2011; Kaczorowski *et al.*, 2013; Dorrington *et al.*, 2015; Harris *et al.*, 2015; Hills *et al.*, 2015; Mader *et al.*, 2016; Marx *et al.*, 2016; Wu *et al.*, 2016; Baxter *et al.*, 2017; Green *et al.*, 2017; Hountz *et al.*, 2017; Weiner *et al.*, 2017; Bakhai *et al.*, 2018; Nguyen *et al.*, 2020; Desai *et al.*, 2021; Funes *et al.*, 2021; Frissora *et al.*, 2021; Hussain *et al.*, 2021; Jones *et al.*, 2022; Ruggeri *et al.*, 2020; Walker-Smith and Baldwin, 2020; Willemse *et al.*, 2022) (Table 3).

The effect of practice-facilitated assessment and improvement, in the form of Quality Improvement (QI) projects and Plan-Do-Study-Act (PDSA) cycles was assessed by sixteen studies. QI projects apply a systematic approach to design, test, and implement interventions (Jones *et al.*, 2019). PDSA cycles are commonly used in QI projects and provide a structured approach to testing interventions and making appropriate adjustments to increase the likelihood of the intervention delivering its’ desired effect.

#### QI projects

Ten practice-facilitated assessment and improvement studies looked at the effect of a QI project on screening. Ruggeri *et al.* (2020) assessed the effect of provider education, patient education, provision of screening kit, and patient reminders. Like Ruggeri *et al.* (2020), Hills *et al.* (2015) reported a positive effect on cervical screening rates following the implementation of a clinical decision support system, provider education, and patient reminders. Walker-Smith and Baldwin (2020) found a positive effect on breast screening following training practice staff to implement screening tools. Frissora *et al.* (2021) looked at the impact of educating and training providers on screening modalities, educating patients, and audit and feedback, Desai *et al.* (2021) assessed the effect of provider education and patient education, and Hussain *et al.* (2021) assessed the impact of provider training, promotional material in the form of a poster, and patient reminder letters. Weiner *et al.* (2017) looked at the effect of a practice facilitator supporting the implementation of office systems, such as screening reminders. Participating practices also received a financial incentive and screening kits to disseminate. All studies had a positive effect on bowel screening participation. Mader *et al.* (2016) evaluated whether education followed by practice-facilitated assessment and improvement, whereby practices worked with QI professionals to conduct activities such as reminder systems streamlining, would increase screening rates. When compared to baseline, a positive effect on bowel screening and breast screening was found, but no effect was found on cervical screening rates. Jones *et al.* (2022) evaluated the effect of audit and feedback and patient reminders on screening rates, finding a positive effect for bowel and cervical; however, no effect for breast. Contrary to the previous practice-facilitated and assessment studies, Nguyen *et al.* (2020) found no effect on bowel, breast, and cervical screening rates when assessing the effect of provider education and financial incentives.

#### Large-scale QI projects

Three studies provided an overview of large-scale QI projects. The QI program utilized PDSA methodology and included components such as provider education and training, and patient education. On a practitioner level, Harris *et al.* (2015) found no effect on bowel screening rates. However, a supplementary population-level study by Green *et al.* (2017) found a positive effect on bowel screening and cervical screening. Marx *et al.* (2016) assessed the effect of a continuous QI project to increase bowel screening rates at five clinics, with components including audit and feedback, provider education and training, provider financial incentives, patient education, and patient reminders. A continuous positive effect was found in three of the five clinics.

#### PDSA cycles

Three studies assessed the effect of PDSA cycles, involving practitioner-focused and patient-focused intervention components. Dorrington *et al.* (2015) used rapid PDSA cycles to implement interventions including education to wider practice-team members and promotional material. A positive effect on cervical screening rates was found. Hountz *et al.* (2017) implemented reminders to nurses, promotional material, and simplified FOBT ordering processes, and Bakhai *et al.* (2018) outlined the effect of eight PDSA cycles, with cycles including interventions such as provider reminders, patient navigation, and patient education. Both studies had a positive effect on bowel screening rates.

#### Practitioner-focused intervention components

Baxter *et al.* (2017) evaluated the impact of strategies on bowel screening uptake, finding practices that employed 4–5 strategies, such as provider reminders and audits and feedback, had a positive effect on bowel screening rates when compared to practices that employed 0–1 strategies.

#### Practitioner-focused and patient-focused intervention components

Five studies involved interventions comprising different combinations of practitioner-focused and patient-focused components. Potter *et al.* (2011) and Funes *et al.* (2021) adapted the FLU-FOBT program. Nurses received education and training on the program, and patients were provided with a screening kit, received education on bowel screening, or a reminder to screen. Wu *et al.* (2016) assessed the effect of an intervention whereby doctors reviewed rosters of patients due for bowel screening and chose practice delegate outreach or default reminder letter. Patients who were referred to the delegate received education about bowel screening and if they declined to undergo colonoscopy, they were facilitated with ordering a FOBT screening kit. In Willemse *et al.* (2022), practice staff received education on the importance of bowel screening and the available options for screening. Patients also received education, along with provider reminders and a screening kit. Kaczorowski *et al.* (2013) assessed the effect on breast and cervical screening rates when combining P4P incentives, provider reminders, and patient reminders. A positive effect was found for all five interventions.

### Contextual factors of interventions

Multi-component studies outlined circumstances under which interventions were more likely to optimize the role of PHCWs and increase participation in screening programs through the use of two strategies: engaging whole teams in a practice setting and the use of champions (Tables 2 and 3). The ‘whole-of-practice approach’ contextual factor is defined as empowering an array of practice staff (eg, administrative staff, nurses, managers, and family physicians) to be involved in cancer screening interventions (Ornstein *et al.*, 2010; Atlas *et al.*, 2011; Shaw *et al.*, 2013; Atlas *et al.*, 2014; Harris *et al.*, 2015; Hills *et al.*, 2015; Mader *et al.*, 2016; Wu *et al.*, 2016; Hountz *et al.*, 2017; Weiner *et al.*, 2017; Bakhai *et al.*, 2018; Nguyen *et al.*, 2020; Hussain *et al.*, 2021; Jones *et al.*, 2022). Ornstein *et al.* (2010) concluded that practices that ‘meet as a team to plan evidence-based (quality) improvement strategies…can achieve much higher levels of screening than typically reported.’ Additionally, a multi-site study by Mader *et al.* (2016) discussed how practices with the greatest change in cancer screening rates ‘had fully engaged staff at several levels within the practice.’ Another contextual factor commonly reported was having a ‘practice champion’ to drive activities within screening programs (Shaw *et al.*, 2013; Hills *et al.*, 2015; Mader *et al.*, 2016; Wu *et al.*, 2016; Weiner *et al.*, 2017; Bakhai *et al.*, 2018; Desai *et al.*, 2021; Jones *et al.*, 2022). Hills *et al.* (2015) reported nearly doubling cervical screening rates (38.1% to 69.7%) following the selection of a practice nurse to facilitate and provide clear direction for a QI project. Similarly, Bakhai *et al.* (2018) outlined that ‘engaged (practice) leadership’ implementing a QI project exceeded their aim of increasing bowel screening rates from a baseline of 50% to 70%, reaching 75%.

## Discussion

This systematic review of 49 studies targeting primary care practices has several key findings as an avenue to increase participation in cancer screening programs. Firstly, interventions with a positive effect were predominantly multi-component, and most included combinations of strategies such as audit and feedback, provider reminders, practice-facilitated assessment and improvement, and patient education across all screening programs. Regarding bowel screening, the provision of screening kits at point-of-care was an effective strategy to increase participation. Secondly, evidence to support the effectiveness of financial incentives for providers was limited, with the review finding most studies to have no effect on screening rates. Finally, ‘whole-of-practice approaches’ and identifying ‘practice champions’ were found to be contextual factors of effective interventions. This study provides novel understanding of components and contextual factors that should be included in interventions using PHCWs to facilitate greater participation in screening programs.

The findings suggest that complex interventions comprised of practitioner-focused and patient-focused components are required to increase cancer screening participation in primary care settings. Supporting the findings of previous research (Emery *et al.*, 2014; Chauhan *et al.*, 2017), audit and feedback were the only intervention components that had a positive effect on cancer screening rates across single-component and multi-component studies. By drawing attention to gaps in performance, audit and feedback likely act as a motivator for PHCWs to change the way they engage with screening programs (Thomson O’Brien *et al.*, 2000). Of the 17 multi-component interventions assessing the effect of practice-facilitated assessments and improvements, fourteen studies reported a positive effect on screening participation rates. Practice-facilitated assessment and improvement may be effective due to being tailored to the needs of the practice, and therefore being more acceptable and appropriate. Existing research suggests provider reminders can increase PHCW’s engagement in cancer screening programs (Emery *et al.*, 2012). Whilst we found provider reminders were often a component of multi-component interventions with a positive effect on screening rates, provider reminders as a single-component intervention did not always have a positive effect on screening rates.

Patient-focused components of interventions were identified as improving the ability of PHCWs to facilitate participation in cancer screening. The benefit of this approach is that it targets different barriers to change (Grimshaw *et al.*, 2001), possibly explaining why provider reminders as a single-component intervention were less effective at increasing screening participation than when incorporated within a multi-component intervention. This is exemplified by Hsiang *et al.* (2019) who found that although provider reminders resulted in a large increase in clinician ordering of tests, there was no effect on bowel and breast screening rates.

Regarding bowel screening, patient education and the provision of a screening kit at point-of-care were common components of effective multi-component interventions. Educating patients before their appointment likely mitigates patient-level barriers to screening, including worry about the procedure or outcome (Ferdous *et al.*, 2018; Suwankhong and Liamputtong, 2018; Muthukrishnan *et al.*, 2019). Additionally, a practitioner providing a bowel cancer screening kit at point-of-care may mitigate structural barriers, such as time (Suwankhong and Liamputtong, 2018; Muthukrishnan *et al.*, 2019), and demonstrates direct endorsement by a PHCW, an important facilitator in a patient’s decision to screen (Duffy *et al.*, 2017). The importance of mitigating structural barriers to screening is highlighted by Potter *et al.* (2011) and Funes *et al.* (2021) who reported a positive effect on bowel screening rates for patients participating in the FLU-FOBT program when compared to non-recipients. A follow-up study by Potter *et al.* (2011) found components of the program and screening rates were maintained one year later (Walsh *et al.*, 2012). Further supporting the importance of making it easier for patients to screen, mailing self-sampling cervical screening kits is more effective at reaching under-screened patients than sending an invitation or reminder letters for clinician sampling (Arbyn *et al.*, 2018; Yeh *et al.*, 2019).

Practitioner-focused financial incentives did not optimize the role of PHCWs, with single-component non-randomized trials reporting no effect on screening participation. However, Jung *et al.* (2017) did find that financial incentives for adopting and using EHRs had a positive effect on screening rates. The difference in these results may be due to financial incentives for engaging patients failing to consider the demanding setting in which PHCWs work, where they are faced with a plethora of competing health issues (Wender, 1993). EHRs facilitate the delivery of care for PHCWs by improving the organization and accessibility of clinical information.

Lastly, our review identified circumstances under which interventions were more likely to optimize the role of PHCWs and increase participation in screening programs. A whole-of-practice approach is likely an effective contextual factor due to spreading practice workload, with ‘time’ a known barrier to PHCWs engaging in cancer screening (Wender, 1993; Yarnall *et al.*, 2003; Verbunt *et al.*, 2022). A whole-of-practice approach is supported by a previous review (Chauhan *et al.*, 2017), which outlined the importance of collaborative team-based interventions to effectively modify PHCW practice and patient outcomes. Having a practice champion to drive cancer screening interventions may also be an important contextual factor due to their role in promoting positive practice culture toward cancer screening programs (Verbunt *et al.*, 2022).

### Limitations

Interventions and study designs were heterogeneous, precluding meta-analysis. It was not possible to present data in forest plots due to the methodological heterogeneity, as well as some studies not reporting *P*-values or confidence intervals. However, our broad inclusion criteria for primary care practice-based interventions and study type (RCTs, non-randomized trials) ensured we captured the range of studies relevant to the topic, which in turn supports the relevance of findings for policy and planning. It is possible that contextual factors relevant to the outcomes were not all described in the original study reports, and have therefore not been acknowledged in this review. Our review only reports on contextual factors that were explicitly outlined in studies surrounding primary care practice-based interventions, with the potential that more implicit contextual factors were missed. Studies were predominantly from the US, and the majority were focused on bowel cancer screening, with findings potentially not generalizable to countries where the structure of the screening program differs.

### Conclusions

Multi-component interventions that are tailored to the needs of a primary care setting, and the patients they serve, may improve the ability of PHCWs to facilitate greater participation in population-based cancer screening programs. Future research should explore the effect of combining identified components of effective interventions (audit and feedback, provider reminders, practice-facilitated assessment and improvement, and patient education across all screening programs and the provision of screening kits at point-of-care for bowel screening) with contextual factors (whole-of-practice approach, practice champion) to maximize screening participation

## Supporting information

Verbunt et al. supplementary material 1 Verbunt et al. supplementary material

Verbunt et al. supplementary material 2 Verbunt et al. supplementary material

## Note

1. The term ‘family physician’ is used to refer to studies conducted in the US and Canada and ‘general practitioners (GPs)’ for studies conducted in all other countries.

## Supplementary material

To view supplementary material for this article, please visit https://doi.org/10.1017/S1463423623000713
