# Supplementary material for: Primary care practice-based interventions and their effect on participation in population-based cancer screening programs: a systematic narrative review
Source: Prim Health Care Res Dev. 2024 Feb 12;25:e12. doi: 10.1017/S1463423623000713 (PMC10894721; doi:10.1017/S1463423623000713)
Supplement: Verbunt et al. supplementary material 1 — Verbunt et al. supplementary material [file S1463423623000713sup001.docx]

| **Single-component RCTs** | | | | | | | |
| --- | --- | --- | --- | --- | --- | --- | --- |
| **Author, Year** | **Aubin-Auger, 2016** | **Dignan, 2014** | **Wang, 2018** | **Guiriguet, 2016** | **Rat, 2017** | **Hwang, 2019** | **Vaisson, 2019** |
| **Reporting** |  | | | | | | |
| 1.Is the hypothesis/aim/objective of the study clearly reported | Yes | Yes | Yes | Yes | Yes | Yes | Yes |
| 2. Are the main outcomes to be measured clearly described in the  Introduction or Methods section? | Yes | Yes | Yes | Yes | Yes | Yes | Yes |
| 3. Are the characteristics of the patients included  in the study clearly described? | Yes | Yes | Yes | Yes | Yes | Yes | Yes |
| 4. Are the interventions of interest clearly described? | Yes | Yes | Yes | Yes | Yes | Yes | Yes |
| 5. Are the distributions of principal confounders in each group of subjects to  be compared clearly described? | Partially | No | Yes (2) | Yes (2) | Partially | Yes (2) | Yes (2) |
| 6. Are the main findings of the study clearly described? | Yes | Yes | Yes | Yes | Yes | Yes | Yes |
| 7. Does the study provide estimates of the random  variability in the data for the main outcomes? | Yes | Yes | Yes | Yes | Yes | Yes | Yes |
| 8. Have all important adverse events that may be  a consequence of the intervention been reported? | No | No | No | No | No | No | No |
| 9. Have the characteristics of patients lost to  follow-up been described? | Yes | No | Yes | Yes | Yes | Yes | Yes |
| 10. Have actual probability values been reported? | Yes | Yes | Yes | Yes | Yes | Yes | Yes |
| **External validity** |  | | | | | | |
| 11. Were the subjects asked to participate in the study representative of the entire population  from which they were recruited? | Yes | Yes | Yes | Yes | Yes | Yes | Yes |
| 12. Were those subjects who were prepared to participate representative of the entire population  from which they were recruited? | Yes | Yes | Yes | Yes | Yes | Yes | Yes |
| 13. Were the staff, places, and facilities where the  patients were treated, representative of the  treatment the majority of patients receive? | Yes | Yes | Yes | Yes | Yes | Yes | Yes |
| **Internal validity – bias** |  | | | | | | |
| 14. Was an attempt made to blind study subjects to  the intervention they have received ? | No | UD | No | No | No | No | Yes |
| 15. Was an attempt made to blind those measuring  the main outcomes of the intervention? | Yes | UD | No | Yes | UD | UD | No |
| 16. If any of the results of the study were based on  “data dredging”, was this made clear? | Yes | Yes | Yes | Yes | Yes | Yes | Yes |
| 17. In trials and cohort studies, do the analyses  adjust for different lengths of follow-up of  patients, or in case-control studies, is the time period between the intervention and outcome  the same for case and controls? | Yes | Yes | Yes | Yes | Yes | Yes | Yes |
| 18. Were the statistical tests used to assess the main outcomes appropriate? | Yes | Yes | Yes | Yes | Yes | Yes | Yes |
| 19. Was compliance with the intervention/s reliable? | UD | UD | UD | UD | UD | UD | UD |
| 20. Were the main outcome measures used  accurate (valid and reliable)? | Yes | Yes | Yes | Yes | Yes | Yes | Yes |
| **Internal validity – confounding (selection bias)** |  | | | | | | |
| 21. Were the patients in different intervention groups (trials and cohort  studies) or were the cases and controls (case‐control studies) recruited from  the same population? | Yes | Yes | Yes | Yes | Yes | Yes | Yes |
| 22. Were study subjects in different intervention groups (trials and cohort  studies) or were the cases and controls (case‐control studies) recruited over the same period of time? | Yes | Yes | Yes | Yes | Yes | Yes | Yes |
| 23. Were study subjects randomised to intervention groups? | Yes | Yes | Yes | Yes | Yes | Yes | Yes |
| 24. Was the randomised intervention assignment concealed from both  patients and health care staff until recruitment was complete and  irrevocable? | No | Yes | Yes | No | UD | No | Yes |
| 25. Was there adequate adjustment for confounding in the analyses from  which the main findings were drawn? | UD | No | Yes | Yes | UD | Yes | Yes |
| 26. Were losses of patients to follow‐up taken into account? | Yes | UD | Yes | Yes | Yes | UD | Yes |
| **Power** |  | | | | | | |
| 27. Did the study have sufficient power to detect a  clinically important effect where the probability value for a difference being due to chance is  less than 5%? | Yes | Yes | Yes | No | Yes | No | Yes |
| **Total score and rating:** | 22 – good | 19 – fair | 24 – good | 23 – good | 21 – good | 21- good | 25 – good |

| **Single-component non-RCTs** | | | | | | | | |
| --- | --- | --- | --- | --- | --- | --- | --- | --- |
| **Author, Year** | **Greene, 2013** | **Kiran, 2014** | **Kirschner, 2013** | **Gavagan, 2010** | **Jung, 2017** | **Curry, 2011** | **Hsiang, 2019** | **Jonah, 2017** |
| **Reporting** |  | | | | | | | |
| 1.Is the hypothesis/aim/objective of the study clearly reported | Yes | Yes | Yes | Yes | Yes | Yes | Yes | Yes |
| 2. Are the main outcomes to be measured clearly described in the  Introduction or Methods section? | Yes | Yes | Yes | Yes | Yes | Yes | Yes | Yes |
| 3. Are the characteristics of the patients included  in the study clearly described? | Yes | Yes | Yes | Yes | Yes | Yes | Yes | Yes |
| 4. Are the interventions of interest clearly described? | Yes | Yes | Yes | Yes | Yes | Yes | Yes | Yes |
| 5. Are the distributions of principal confounders in each group of subjects to  be compared clearly described? | No | Yes (2) | No | No | Yes (2) | No | Yes (2) | Yes |
| 6. Are the main findings of the study clearly described? | Yes | Yes | Yes | Yes | Yes | Yes | Yes | No |
| 7. Does the study provide estimates of the random  variability in the data for the main outcomes? | Yes | Yes | Yes | Yes | Yes | Yes | Yes | Yes |
| 8. Have all important adverse events that may be  a consequence of the intervention been reported? | Yes | No | No | No | No | No | No | No |
| 9. Have the characteristics of patients lost to  follow-up been described? | No | No | No | No | No | No | No | No |
| 10. Have actual probability values been reported? | Yes | Yes | Yes | Yes | Yes | Yes | Yes | No |
| **External validity** |  | | | | | | | |
| 11. Were the subjects asked to participate in the study representative of the entire population  from which they were recruited? | Yes | Yes | Yes | Yes | Yes | Yes | Yes | Yes |
| 12. Were those subjects who were prepared to participate representative of the entire population  from which they were recruited? | Yes | Yes | Yes | Yes | Yes | Yes | Yes | Yes |
| 13. Were the staff, places, and facilities where the  patients were treated, representative of the  treatment the majority of patients receive? | Yes | Yes | Yes | Yes | Yes | Yes | Yes | Yes |
| **Internal validity – bias** |  | | | | | | | |
| 14. Was an attempt made to blind study subjects to  the intervention they have received ? | No | No | No | No | No | No | No | No |
| 15. Was an attempt made to blind those measuring  the main outcomes of the intervention? | No | No | UD | No | No | No | UD | No |
| 16. If any of the results of the study were based on  “data dredging”, was this made clear? | Yes | Yes | Yes | Yes | Yes | Yes | Yes | Yes |
| 17. In trials and cohort studies, do the analyses  adjust for different lengths of follow-up of  patients, or in case-control studies, is the time period between the intervention and outcome  the same for cases and controls? | Yes | Yes | Yes | Yes | Yes | Yes | Yes | Yes |
| 18. Were the statistical tests used to assess the main outcomes appropriate? | Yes | Yes | Yes | Yes | Yes | Yes | Yes | Yes |
| 19. Was compliance with the intervention/s reliable? | UD | UD | UD | UD | UD | UD | UD | Yes |
| 20. Were the main outcome measures used  accurate (valid and reliable)? | Yes | Yes | Yes | Yes | Yes | Yes | Yes | Yes |
| **Internal validity – confounding (selection bias)** |  | | | | | | | |
| 21. Were the patients in different intervention groups (trials and cohort  studies) or were the cases and controls (case‐control studies) recruited from  the same population? | Yes | Yes | Yes | Yes | Yes | Yes | Yes | Yes |
| 22. Were study subjects in different intervention groups (trials and cohort  studies) or were the cases and controls (case‐control studies) recruited over the same period of time? | Yes | Yes | Yes | Yes | Yes | Yes | Yes | Yes |
| 23. Were study subjects randomised to intervention groups? | No | No | Yes | No | No | No | No | No |
| 24. Was the randomised intervention assignment concealed from both  patients and health care staff until recruitment was complete and  irrevocable? | No | No | Yes | No | No | No | No | No |
| 25. Was there adequate adjustment for confounding in the analyses from  which the main findings were drawn? | No | Yes | No | No | Yes | No | Yes | Yes |
| 26. Were losses of patients to follow‐up taken into account? | UD | UD | UD | No | UD | UD | UD | UD |
| **Power** |  | | | | | | | |
| 27. Did the study have sufficient power to detect a  clinically important effect where the probability value for a difference being due to chance is  less than 5%? | No | No | No | Yes | No | No | No | No |
| **Total score and rating:** | 16 – fair | 19 – fair | 18 – fair | 17 – fair | 18 – fair | 16 – fair | 19 – fair | 18 – fair |

| **Multi-component RCTs** | | | | | | | | | | | | |
| --- | --- | --- | --- | --- | --- | --- | --- | --- | --- | --- | --- | --- |
| **Author, Year** | **Price-Haywood, 2014** | **Shaw, 2013** | **Ornstien, 2010** | **Aragones, 2010** | **Moen, 2010** | **Walsh, 2020** | **Atlas, 2011** | **Atlas, 2014** | **Dodd, 2019** | **Sun, 2018** | **Basch, 2015** | **Cameron, 2020** |
| **Reporting** |  | | | | | | | | | | | |
| 1.Is the hypothesis/aim/objective of the study clearly reported | Yes | Yes | Yes | Yes | Yes | Yes | Yes | Yes | Yes | Yes | Yes | Yes |
| 2. Are the main outcomes to be measured clearly described in the  Introduction or Methods section? | Yes | Yes | Yes | Yes | Yes | Yes | Yes | Yes | Yes | Yes | Yes | Yes |
| 3. Are the characteristics of the patients included  in the study clearly described? | Yes | Yes | Yes | Yes | Yes | Yes | Yes | Yes | Yes | Yes | Yes | Yes |
| 4. Are the interventions of interest clearly described? | Yes | Yes | Yes | Yes | Yes | Yes | Yes | Yes | Yes | Yes | Yes | Yes |
| 5. Are the distributions of principal confounders in each group of subjects to  be compared clearly described? | Yes (2) | Yes (2) | Yes (2) | Yes (2) | Yes (2) | Yes (2) | Yes (2) | Yes (2) | No | No | Yes (2) | Yes (2) |
| 6. Are the main findings of the study clearly described? | Yes | Yes | Yes | Yes | Yes | Yes | Yes | Yes | Yes | Yes | Yes | Yes |
| 7. Does the study provide estimates of the random  variability in the data for the main outcomes? | Yes | Yes | Yes | Yes | Yes | Yes | Yes | Yes | Yes | Yes | Yes | Yes |
| 8. Have all important adverse events that may be  a consequence of the intervention been reported? | No | Yes | No | No | No | No | No | No | No | No | No | No |
| 9. Have the characteristics of patients lost to  follow-up been described? | Yes | Yes | Yes | Yes | No | No | No | Yes | Yes | No | Yes | Yes |
| 10. Have actual probability values been reported? | Yes | Yes | Yes | Yes | Yes | Yes | Yes | Yes | Yes | Yes | Yes | Yes |
| **External validity** |  | | | | | | | | | | | |
| 11. Were the subjects asked to participate in the study representative of the entire population  from which they were recruited? | Yes | Yes | Yes | Yes | Yes | Yes | Yes | Yes | Yes | Yes | Yes | Yes |
| 12. Were those subjects who were prepared to participate representative of the entire population  from which they were recruited? | Yes | Yes | Yes | Yes | Yes | Yes | Yes | Yes | Yes | Yes | Yes | Yes |
| 13. Were the staff, places, and facilities where the  patients were treated, representative of the  treatment the majority of patients receive? | Yes | Yes | Yes | Yes | Yes | Yes | Yes | Yes | Yes | Yes | Yes | Yes |
| **Internal validity – bias** |  | | | | | | | | | | | |
| 14. Was an attempt made to blind study subjects to  the intervention they have received ? | Yes | No | No | No | No | No | No | No | No | UD | No | No |
| 15. Was an attempt made to blind those measuring  the main outcomes of the intervention? | No | No | No | Yes | UD | Yes | No | No | UD | UD | UD | UD |
| 16. If any of the results of the study were based on  “data dredging”, was this made clear? | No | Yes | Yes | Yes | Yes | Yes | Yes | Yes | Yes | Yes | Yes | Yes |
| 17. In trials and cohort studies, do the analyses  adjust for different lengths of follow-up of  patients, or in case-control studies, is the time period between the intervention and outcome  the same for cases and controls? | Yes | Yes | Yes | Yes | Yes | Yes | Yes | Yes | Yes | Yes | Yes | Yes |
| 18. Were the statistical tests used to assess the main outcomes appropriate? | Yes | Yes | Yes | Yes | Yes | Yes | Yes | Yes | Yes | Yes | Yes | Yes |
| 19. Was compliance with the intervention/s reliable? | UD | UD | UD | UD | UD | UD | UD | UD | UD | UD | UD | UD |
| 20. Were the main outcome measures used  accurate (valid and reliable)? | Yes | Yes | Yes | Yes | Yes | Yes | Yes | Yes | Yes | Yes | Yes | Yes |
| **Internal validity – confounding (selection bias)** |  | | | | | | | | | | | |
| 21. Were the patients in different intervention groups (trials and cohort  studies) or were the cases and controls (case‐control studies) recruited from  the same population? | Yes | Yes | Yes | Yes | Yes | Yes | Yes | Yes | Yes | Yes | Yes | Yes |
| 22. Were study subjects in different intervention groups (trials and cohort  studies) or were the cases and controls (case‐control studies) recruited over the same period of time? | Yes | Yes | Yes | Yes | Yes | Yes | Yes | Yes | Yes | Yes | Yes | Yes |
| 23. Were study subjects randomised to intervention groups? | Yes | Yes | Yes | Yes | Yes | Yes | Yes | Yes | Yes | Yes | Yes | Yes |
| 24. Was the randomised intervention assignment concealed from both  patients and health care staff until recruitment was complete and  irrevocable? | No | No | No | Yes | No | UD | No | Yes | No | No | No | No |
| 25. Was there adequate adjustment for confounding in the analyses from  which the main findings were drawn? | Yes | Yes | Yes | Yes | UD | Yes | Yes | Yes | UD | UD | Yes | Yes |
| 26. Were losses of patients to follow‐up taken into account? | Yes | Yes | Yes | Yes | UD | UD | UD | Yes | Yes | UD | Yes | Yes |
| **Power** |  | | | | | | | | | | | |
| 27. Did the study have sufficient power to detect a  clinically important effect where the probability value for a difference being due to chance is  less than 5%? | No | Yes | Yes | No | Yes | No | Yes | Yes | Yes | No | No | Yes |
| **Total score and rating:** | 22 – good | 24 – good | 23 – good | 24 – good | 20 – good | 21 – good | 21 – good | 24 – good | 20 – good | 17 – fair | 22 – good | 23 - good |

| **Multi-component non-RCTs** | | | | | | | | | | | |
| --- | --- | --- | --- | --- | --- | --- | --- | --- | --- | --- | --- |
| **Author, Year** | **Baxter, 2017** | **Dorrington, 2015** | **Hountz, 2017** | **Bakhai, 2018** | **Ruggeri, 2020** | **Hills, 2015** | **Walker-Smith, 2020** | **Frissora, 2021** | **Mader, 2021** | **Desai, 2021** | **Hussain, 2021** |
| **Reporting** |  | | | | | | | | | | |
| 1.Is the hypothesis/aim/objective of the study clearly reported | Yes | Yes | Yes | Yes | Yes | Yes | Yes | Yes | Yes | Yes | Yes |
| 2. Are the main outcomes to be measured clearly described in the  Introduction or Methods section? | Yes | Yes | Yes | Yes | Yes | Yes | Yes | Yes | Yes | Yes | Yes |
| 3. Are the characteristics of the patients included  in the study clearly described? | Yes | Yes | Yes | Yes | Yes | Yes | Yes | Yes | Yes | Yes | Yes |
| 4. Are the interventions of interest clearly described? | Yes | Yes | Yes | Yes | Yes | Yes | Yes | Yes | Yes | Yes | Yes |
| 5. Are the distributions of principal confounders in each group of subjects to  be compared clearly described? | Yes (2) | No | Partially | No | No | No | No | No | No | No | Yes (2) |
| 6. Are the main findings of the study clearly described? | Yes | Yes | Yes | Yes | Yes | Yes | Yes | Yes | Yes | Yes | Yes |
| 7. Does the study provide estimates of the random  variability in the data for the main outcomes? | Yes | Yes | Yes | Yes | No | Yes | Yes | Yes | Yes | Yes | Yes |
| 8. Have all important adverse events that may be  a consequence of the intervention been reported? | No | Yes | No | Yes | Yes | No | No | No | Yes | No | No |
| 9. Have the characteristics of patients lost to  follow-up been described? | Yes | Yes | No | No | No | No | No | No | No | No | No |
| 10. Have actual probability values been reported? | Yes | Yes | No | No | No | Yes | Yes | Yes | Yes | Yes | Yes |
| **External validity** |  | | | | | | | | | | |
| 11. Were the subjects asked to participate in the study representative of the entire population  from which they were recruited? | Yes | Yes | Yes | Yes | Yes | Yes | Yes | Yes | Yes | Yes | Yes |
| 12. Were those subjects who were prepared to participate representative of the entire population  from which they were recruited? | Yes | Yes | UD | Yes | Yes | Yes | Yes | Yes | Yes | Yes | Yes |
| 13. Were the staff, places, and facilities where the  patients were treated, representative of the  treatment the majority of patients receive? | Yes | Yes | Yes | Yes | Yes | Yes | Yes | Yes | Yes | Yes | No |
| **Internal validity – bias** |  | | | | | | | | | | |
| 14. Was an attempt made to blind study subjects to  the intervention they have received ? | No | No | No | No | No | No | No | No | No | No | No |
| 15. Was an attempt made to blind those measuring  the main outcomes of the intervention? | UD | No | No | No | No | No | No | No | No | No | No |
| 16. If any of the results of the study were based on  “data dredging”, was this made clear? | Yes | Yes | Yes | Yes | Yes | Yes | Yes | Yes | Yes | Yes | Yes |
| 17. In trials and cohort studies, do the analyses  adjust for different lengths of follow-up of  patients, or in case-control studies, is the time period between the intervention and outcome  the same for cases and controls? | Yes | Yes | Yes | Yes | Yes | Yes | Yes | Yes | Yes | Yes | Yes |
| 18. Were the statistical tests used to assess the main outcomes appropriate? | Yes | Yes | Yes | Yes | Yes | No | Yes | Yes | Yes | Yes | Yes |
| 19. Was compliance with the intervention/s reliable? | UD | UD | UD | UD | UD | UD | UD | UD | UD | UD | UD |
| 20. Were the main outcome measures used  accurate (valid and reliable)? | Yes | Yes | Yes | Yes | Yes | Yes | Yes | Yes | Yes | Yes | Yes |
| **Internal validity – confounding (selection bias)** |  | | | | | | | | | | |
| 21. Were the patients in different intervention groups (trials and cohort  studies) or were the cases and controls (case‐control studies) recruited from  the same population? | Yes | Yes | Yes | Yes | Yes | Yes | Yes | Yes | Yes | Yes | Yes |
| 22. Were study subjects in different intervention groups (trials and cohort  studies) or were the cases and controls (case‐control studies) recruited over the same period of time? | Yes | Yes | Yes | Yes | Yes | Yes | Yes | Yes | Yes | Yes | Yes |
| 23. Were study subjects randomised to intervention groups? | Yes | No | No | No | No | No | No | No | No | No | No |
| 24. Was the randomised intervention assignment concealed from both  patients and health care staff until recruitment was complete and  irrevocable? | No | No | No | No | No | No | No | No | No | No | No |
| 25. Was there adequate adjustment for confounding in the analyses from  which the main findings were drawn? | UD | No | No | No | No | UD | UD | No | No | No | Yes |
| 26. Were losses of patients to follow‐up taken into account? | UD | UD | Yes | UD | UD | UD | UD | UD | UD | UD | UD |
| **Power** |  | | | | | | | | | | |
| 27. Did the study have sufficient power to detect a  clinically important effect where the probability value for a difference being due to chance is  less than 5%? | Yes | No | No | No | No | No | No | No | No | No | No |
| **Total score and rating:** | 19 – fair | 18 – fair | 16 – fair | 16 – fair | 15 – fair | 16 – fair | 16 – fair | 16 – fair | 17 – fair | 16 – fair | 18 – fair |

| **Multi-component non-RCTs (continued)** | | | | | | | | | | | |
| --- | --- | --- | --- | --- | --- | --- | --- | --- | --- | --- | --- |
| **Author, Year** | **Jones, 2022** | **Marx, 2016** | **Nguyen, 2020** | **Harris, 2014** | **Green, 2017** | **Weiner, 2017** | **Potter, 2011** | **Funes, 2021** | **Wu, 2016** | **Kaczorowski, 2013** | **Willemse, 2022** |
| **Reporting** |  | | | | | | | |  |  |  |
| 1.Is the hypothesis/aim/objective of the study clearly reported | Yes | Yes | Yes | Yes | Yes | Yes | Yes | Yes | Yes | Yes | Yes |
| 2. Are the main outcomes to be measured clearly described in the  Introduction or Methods section? | Yes | Yes | Yes | Yes | Yes | Yes | Yes | Yes | Yes | Yes | Yes |
| 3. Are the characteristics of the patients included  in the study clearly described? | Yes | Yes | Yes | Yes | Yes | Yes | Yes | Yes | Yes | Yes | Yes |
| 4. Are the interventions of interest clearly described? | Yes | Yes | Yes | Yes | Yes | Yes | Yes | Yes | Yes | Yes | Yes |
| 5. Are the distributions of principal confounders in each group of subjects to  be compared clearly described? | Yes (2) | No | No | No | Yes (2) | No | No | UD | Yes (2) | No | Partially |
| 6. Are the main findings of the study clearly described? | Yes | Yes | Yes | Yes | Yes | Yes | Yes | Yes | Yes | Yes | Yes |
| 7. Does the study provide estimates of the random  variability in the data for the main outcomes? | Yes | Yes | Yes | Yes | Yes | Yes | Yes | Yes | Yes | Yes | No |
| 8. Have all important adverse events that may be  a consequence of the intervention been reported? | No | No | No | Yes | No | No | No | No | No | No | No |
| 9. Have the characteristics of patients lost to  follow-up been described? | No | No | No | No | Yes | No | No | No | No | Yes | No |
| 10. Have actual probability values been reported? | Yes | Yes | Yes | Yes | Yes | Yes | Yes | Yes | Yes | Yes | No |
| **External validity** |  | | | | | | | | | | |
| 11. Were the subjects asked to participate in the study representative of the entire population  from which they were recruited? | Yes | Yes | Yes | Yes | Yes | Yes | Yes | Yes | Yes | Yes | Yes |
| 12. Were those subjects who were prepared to participate representative of the entire population  from which they were recruited? | Yes | Yes | UD | Yes | Yes | Yes | Yes | Yes | Yes | Yes | Yes |
| 13. Were the staff, places, and facilities where the  patients were treated, representative of the  treatment the majority of patients receive? | Yes | Yes | Yes | Yes | Yes | Yes | Yes | Yes | Yes | Yes | Yes |
| **Internal validity – bias** |  | | | | | | | | | | |
| 14. Was an attempt made to blind study subjects to  the intervention they have received ? | No | No | No | No | No | No | No | No | No | No | No |
| 15. Was an attempt made to blind those measuring  the main outcomes of the intervention? | No | UD | UD | UD | UD | UD | No | No | No | UD | No |
| 16. If any of the results of the study were based on  “data dredging”, was this made clear? | Yes | Yes | Yes | Yes | Yes | Yes | Yes | Yes | Yes | Yes | Yes |
| 17. In trials and cohort studies, do the analyses  adjust for different lengths of follow-up of  patients, or in case-control studies, is the time period between the intervention and outcome  the same for cases and controls? | Yes | Yes | Yes | Yes | Yes | Yes | Yes | Yes | Yes | Yes | Yes |
| 18. Were the statistical tests used to assess the main outcomes appropriate? | Yes | Yes | Yes | Yes | Yes | Yes | Yes | Yes | Yes | Yes | Yes |
| 19. Was compliance with the intervention/s reliable? | UD | UD | UD | UD | UD | UD | UD | UD | UD | UD | UD |
| 20. Were the main outcome measures used  accurate (valid and reliable)? | Yes | Yes | Yes | Yes | Yes | Yes | Yes | Yes | Yes | Yes | Yes |
| **Internal validity – confounding (selection bias)** |  | | | | | | | | | | |
| 21. Were the patients in different intervention groups (trials and cohort  studies) or were the cases and controls (case‐control studies) recruited from  the same population? | Yes | Yes | Yes | Yes | Yes | Yes | Yes | Yes | Yes | Yes | Yes |
| 22. Were study subjects in different intervention groups (trials and cohort  studies) or were the cases and controls (case‐control studies) recruited over the same period of time? | Yes | Yes | Yes | Yes | Yes | Yes | Yes | Yes | Yes | Yes | No |
| 23. Were study subjects randomised to intervention groups? | No | Yes | No | Yes | Yes | No | No | No | No | No | No |
| 24. Was the randomised intervention assignment concealed from both  patients and health care staff until recruitment was complete and  irrevocable? | No | No | No | Yes | No | No | No | No | No | No | No |
| 25. Was there adequate adjustment for confounding in the analyses from  which the main findings were drawn? | Yes | UD | No | No | UD | Yes | No | No | No | UD | Partially |
| 26. Were losses of patients to follow‐up taken into account? | UD | UD | UD | UD | UD | UD | UD | No | UD | Yes | No |
| **Power** |  | | | | | | | | | | |
| 27. Did the study have sufficient power to detect a  clinically important effect where the probability value for a difference being due to chance is  less than 5%? | No | No | No | Yes | No | No | No | No | No | No | No |
| **Total score and rating:** | 19 – fair | 16 - fair | 15 – fair | 20 – good | 20 – good | 17 – fair | 16 – fair | 16 – fair | 17 – fair | 18 – fair | 15 – fair |
