# Supplementary material for: Primary care practice-based interventions and their effect on participation in population-based cancer screening programs: a systematic narrative review
Source: Prim Health Care Res Dev. 2024 Feb 12;25:e12. doi: 10.1017/S1463423623000713 (PMC10894721; doi:10.1017/S1463423623000713)
Supplement: Verbunt et al. supplementary material 2 — Verbunt et al. supplementary material [file S1463423623000713sup002.docx]

**Appendix 1. Search strategy**

**Medline (OVID):**

1     ((general practi* or physician* or primary care or primary healthcare or primary health care or doctor* or nurse practitioner* or primary health or Aboriginal medical service) adj4 (intervention* or program* or strateg* or initiative* or tool* or approach* or education*)).mp.

2     ((cervix or cervical or breast or bowel or colorectal or colon*) adj4 (cancer* or neoplasm or tumour* or tumor* or carcinoma*)).mp.

3     (screen* or "early detection" or depistage or pap or Papanicolaou or HPV or mammography or mammogram or self-test* or faecal occult blood test* or fecal occult blood test* or FOBT or faecal immunochemical test* or fecal immunochemical test* or FIT or immunochemical faecal occult blood test* or immunochemical fecal occult blood test or IFOBT).mp.

4     1 and 2 and 3 (679)

5     limit 4 to yr="2010 - 2022"

**EMBASE:**

1     ((general practi* or physician* or primary care or primary healthcare or primary health care or doctor* or nurse practitioner* or primary health or Aboriginal medical service) adj4 (intervention* or program* or strateg* or initiative* or tool* or approach* or education*)).mp. (60015)

2     ((cervix or cervical or breast or bowel or colorectal or colon*) adj4 (cancer* or neoplasm or tumour* or tumor* or carcinoma*)).mp. (1070827)

3     (screen* or "early detection" or depistage or pap or Papanicolaou or HPV or mammography or mammogram or self-test* or faecal occult blood test* or fecal occult blood test* or FOBT or faecal immunochemical test* or fecal immunochemical test* or FIT or immunochemical faecal occult blood test* or immunochemical fecal occult blood test or IFOBT).mp. (1694808)

4     1 and 2 and 3

5     limit 4 to yr="2010 – 2022

6 limit 5 to (article or article in press)

7 limit 6 to exclude Medline journals

**Cumulative Index to Nursing and Allied Health Literature (CINAHL):**

("general practi* or physician*" or "primary care" or "primary healthcare" or "primary health care" or doctor* or "nurse practitioner*" or "primary health" or "Aboriginal medical service") N4 (intervention* or program* or strateg* or initiative* or tool* or approach* or education*)

(cervix or cervical or breast or bowel or colorectal or colon*) N4 (cancer* or neoplasm or tumour* or tumor* or carcinoma*)

screen* or "early detection" or depistage or pap or Papanicolaou or HPV or mammography or mammogram or self-test* or "faecal occult blood test*" or "fecal occult blood test*" or FOBT or "faecal immunochemical test*" or "fecal immunochemical test*" or FIT or "immunochemical faecal occult blood test*" or "immunochemical fecal occult blood test" or IFOBT

Limit to (Publication Date) – 2010-2022

Source Types – Academic Journals
